# Monitoring Protein Misfolding by Site-Specific Labeling of Proteins *In Vivo*

**DOI:** 10.1371/journal.pone.0099395

**Published:** 2014-06-10

**Authors:** Tzung-yang Hsieh, Nadinath B. Nillegoda, Jens Tyedmers, Bernd Bukau, Axel Mogk, Günter Kramer

**Affiliations:** Zentrum für Molekulare Biologie der Universität Heidelberg (ZMBH), Deutsches Krebsforschungszentrum (DKFZ), DKFZ-ZMBH Alliance, Heidelberg, Germany; University of Geneva, Switzerland

## Abstract

Incorporating fluorescent amino acids by suppression of the TAG amber codon is a useful tool for site-specific labeling of proteins and visualizing their localization in living cells. Here we use a plasmid encoded orthogonal tRNA/aminoacyl-tRNA synthetase pair to site-specifically label firefly luciferase with the environmentally sensitive fluorescent amino acid, 3-(6-acetylnaphthalen-2-ylamino)-2- aminopropanoic acid (ANAP) and explore the detectability of conformational changes in labeled luciferase in the yeast cytoplasm. We find that ANAP labeling efficiency is greatly increased in [*PSI* ^+^] cells and show that analysis of the ANAP fluorescence emission by confocal imaging allows for tracking the thermal unfolding and aggregation of luciferase *in vivo*. Furthermore we demonstrate that flow cytometry can be used to study conformational changes in luciferase and chaperone-mediated refolding in quantitative terms and at the level of single cells. This experimental setup for the first time allows for the direct analysis of the folding state of a protein in living cells and may serve as valuable new tool for examining mechanisms of protein folding, misfolding and aggregation.

## Introduction

Protein folding often initiates during synthesis at ribosomes as the linear polypeptide enters the crowded cytoplasm [1]. Once folded, the energy barriers between folded and misfolded states are often rather small, putting proteins at constant risk of misfolding. Causes for misfolding are numerous, ranging from impairment of native state stability by mutations or translation errors, to misfolding through cellular ageing or the exposure to environmental stress conditions such as heat or oxidative stress [2]. To protect the proteome from misfolding and aggregation, cells exploit protein quality maintenance and surveillance systems with different activities ranging from prevention of misfolding, refolding of misfolded species, disaggregation of protein aggregates to the controlled degradation of terminally misfolded species [3]. Exhaustion of the quality control system causes the accumulation of misfolded species and eventually the formation of potentially toxic protein aggregates. Protein aggregates have been linked to various human diseases ranging from cancer and type 2 diabetes to neurodegenerative diseases, such as Alzheimer's disease, Parkinson's disease, Huntington disease and Amyotrophic lateral sclerosis [4], [5].

For dissecting the mechanisms of protein folding/misfolding and aggregate formation at the molecular level, tools that allow for capturing the dynamics of conformational changes *in vivo* are of great interest. Current approaches include the analysis of protein activity or protein solubility in cell lysates or the microscopic analysis of aggregate formation of the proteins fused either N- or C-terminally to fluorescent proteins. However, the large size of these fluorescent tags (>20 kDa) often interferes with the function and potentially also the localization of the protein of interest [6]. Furthermore, since misfolding is detectable only upon accumulation of proteins in large aggregates, small misfolded proteins or protein aggregates potentially escape the detection. An alternative approach for fluorescent labeling of proteins employs plasmid encoded orthogonal tRNA/aminoacyl-tRNA synthetase (aaRS) pairs that can be expressed in cells to suppress non-sense codons in protein coding sequences resulting in the specific incorporation of unnatural amino acids in any target protein of choice. The repertoire of such incorporation systems is rapidly growing and includes a large number of structurally und functionally distinct unnatural amino acids that can be incorporated in many different organisms including bacteria, yeast and mammalian cells [7].

Here, we describe that site-specific labeling of proteins with the fluorescent amino acid 3-(6-acetylnaphthalen-2-ylamino)-2-aminopropanoic acid (ANAP) can be used for the analysis of both protein localization and the protein folding state in *Saccharomyces cerevisiae in vivo*. Using the well characterized thermolabile *Photinus pyralis* luciferase as a model substrate, we demonstrate that heat-induced unfolding, aggregation and chaperone-dependent refolding of protein can be monitored by analysis of the fluorescence emission spectra of the labeled protein in time-resolved manner using flow cytometry or confocal microscopy and wavelength scans. Finally we show that the efficiency of TAG amber codon suppression labeling in yeast cells is greatly facilitated by reduced translation termination efficiency in [*PSI* ^+^] cells or in cells containing reduced levels of the Sup35 protein. This new application for site-specific labeling of proteins sets the stage for a more comprehensive analysis of protein conformational changes in the natural context of a living cell and should be readily applicable to other protein substrates and cell types including mammalian cells.

## Results

### Site-specific Incorporation of ANAP into firefly luciferase

For the analysis of protein conformational changes *in vivo* we were seeking a fluorescent probe that combines several features: (i) integration should be site-specific with minimal perturbation of protein structure and function, (ii) labeling should be possible in a variety of eukaryotic cells, (iii) the fluorescent dye should be bright and (iv) the dye should be environmentally sensitive, i.e. exhibit a significant shift in the emission maximum upon change in the solvent polarity. A fluorescent probe that fulfills all these requirements is 3-(6-acetylnaphthalen-2-ylamino)-2-aminopropanoicacid, (ANAP), an unnatural amino acid derivative of prodan (6-propionyl-2-(N,N-dimethyl)- aminonaphthalene) [8]. The maximum emission wavelength of ANAP, like that of the prodan moiety, greatly depends on the local environment and ranges between 490 nm in water and 420 nm in ethyl acetate [9].

ANAP incorporation is possible in both yeast [9] and human cells [10]. As a proof of principle we chose the well-characterized firefly luciferase that readily denatures and aggregates upon heat exposure in yeast cells. In wild-type cells and upon relief of heat stress the aggregated luciferase can be refolded by the AAA+ protein Hsp104 in cooperation with Hsp70 and Hsp40 chaperones [11]. Misfolding, aggregation and refolding of luciferase *in vivo* can be detected by measuring the luciferase-catalyzed oxidation of luciferin to oxyluciferin that results in bioluminescence.

**Figure 1:**
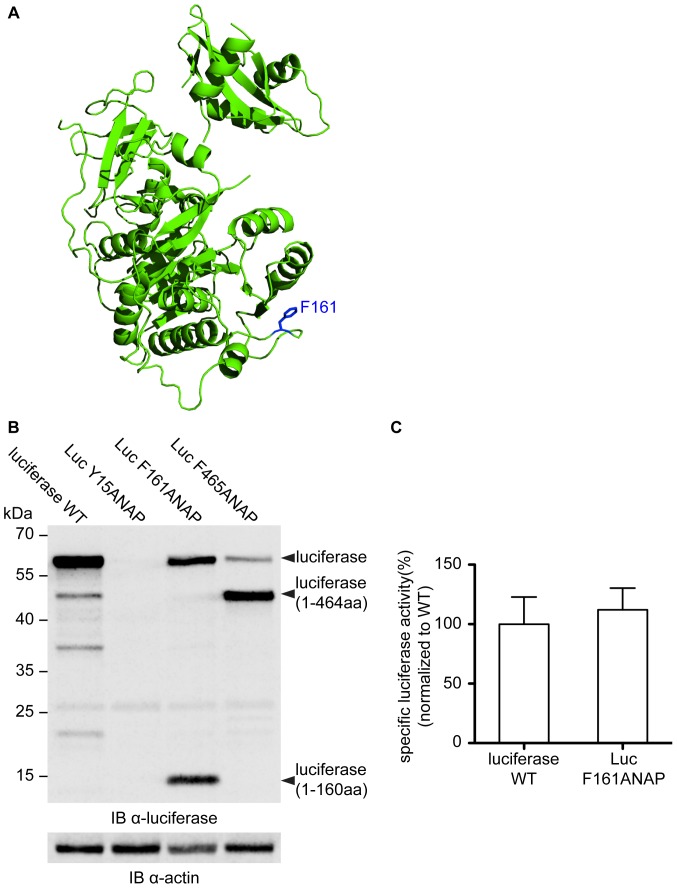
Luciferase mutant F161ANAP retains wild-type like activity. Crystal structure of the *Photinus pyralis* firefly luciferase (PDB ID = 1LCI). Phenylalanine 161 mutated in Luc F161ANAP for the incorporation of 3-(6-acetylnaphthalen-2-ylamino)-2-aminopropanoic acid (ANAP) is highlighted in blue. (A) Gel analysis of wild-type and ANAP-incorporated luciferase variants. Expression level of luciferase and mutant derivatives was analyzed by immunoblotting with antisera specific for firefly luciferase. Actin specific antiserum (α-Actin) was used as a loading control. Luciferase (1-464aa) and Luciferase (1-160aa) represent N-terminal fragments derived from translation termination at the TAG amber codon introduced at the respective codon of the mutated luciferase genes. (B) Specific activity of Luc F161ANAP normalized to the specific activity of wild-type luciferase. The specific activity of wild-type luciferase was set to 100%. Specific activities were calculated as the ratio of the measured luciferase activity and the steady-state luciferase level (as determined by quantitative western blotting).

We started our analysis by screening for a position where ANAP incorporation does not affect luciferase folding and expression. We limited our selection to tyrosine, tryptophan or phenylalanine residues, which are structurally most similar to ANAP. Some of these mutated amino acids were surface exposed while others were buried in the folded protein (Figure 1A, Table S1 in File S1). A total of eight mutant genes were created and analyzed *in vivo* by determining the luciferase activity after overexpression for seven hours. We detected a significant variation in luciferase activity ranging from 0.02% to 26% of the wild-type luciferase activity (Table S1 in File S1). In most cases this low luciferase activity was due to the inefficient suppression of the amber stop codon that results in the synthesis of an N-terminal fragment of luciferase (Figure 1B and data not shown). The by far best expression was observed for the mutant luciferase Luc F161ANAP in which the prodan fluorophore is positioned on a loop at the protein surface (Figure 1A). Since the Luc F161ANAP specific activity was very similar to that of wildtype luciferase demonstrating native-like folding (Figure 1C), we focused on Luc F161ANAP for all subsequent experiments.

### ANAP incorporation efficiency can be increased by reduction of Sup35 levels in [PSI^+^] cells

Initial ANAP incorporation experiments revealed limited TAG suppression efficiency even for Luc F161ANAP, which was evident by the accumulation of ∼18 kDa protein fragment consisting of the first 160 amino acids of luciferase (Figure 1B). We rationalized that this problem might be overcome by reducing the efficiency of translation termination. A rather simple possibility to achieve this is the use of yeast strains conferring the [*PSI* ^+^] phenotype. This phenotype is caused by the sequestration of parts of the Sup35 protein in prion aggregates thereby reducing the concentration of soluble active Sup35. Since Sup35 together with Sup45 constitutes the eukaryotic translation release factor [12], [*PSI* ^+^] cells exhibit an enhanced frequency of translational read-through of stop codons. To test this hypothesis, we compared Luc F161ANAP expression in strain OT56 conferring either the [*psi* ^−^] or the [*PSI* ^+^] phenotype [13]. Indeed we observed a drastic increase in the amount of Luc F161ANAP full-length protein in [*PSI* ^+^] compared to [*psi* ^−^] cells (Figure S1A). Furthermore, after seven hours of Luc F161ANAP expression in the presence of the unnatural amino acid, [*PSI* ^+^] cells showed much stronger ANAP fluorescence than OT56 [*psi* ^−^] cells (Figure S1B). Therefore, unless specified otherwise, the [*PSI* ^+^] strain OT56 was used for subsequent experiment.

### Specific labeling of Luciferase with ANAP

**Figure 2:**
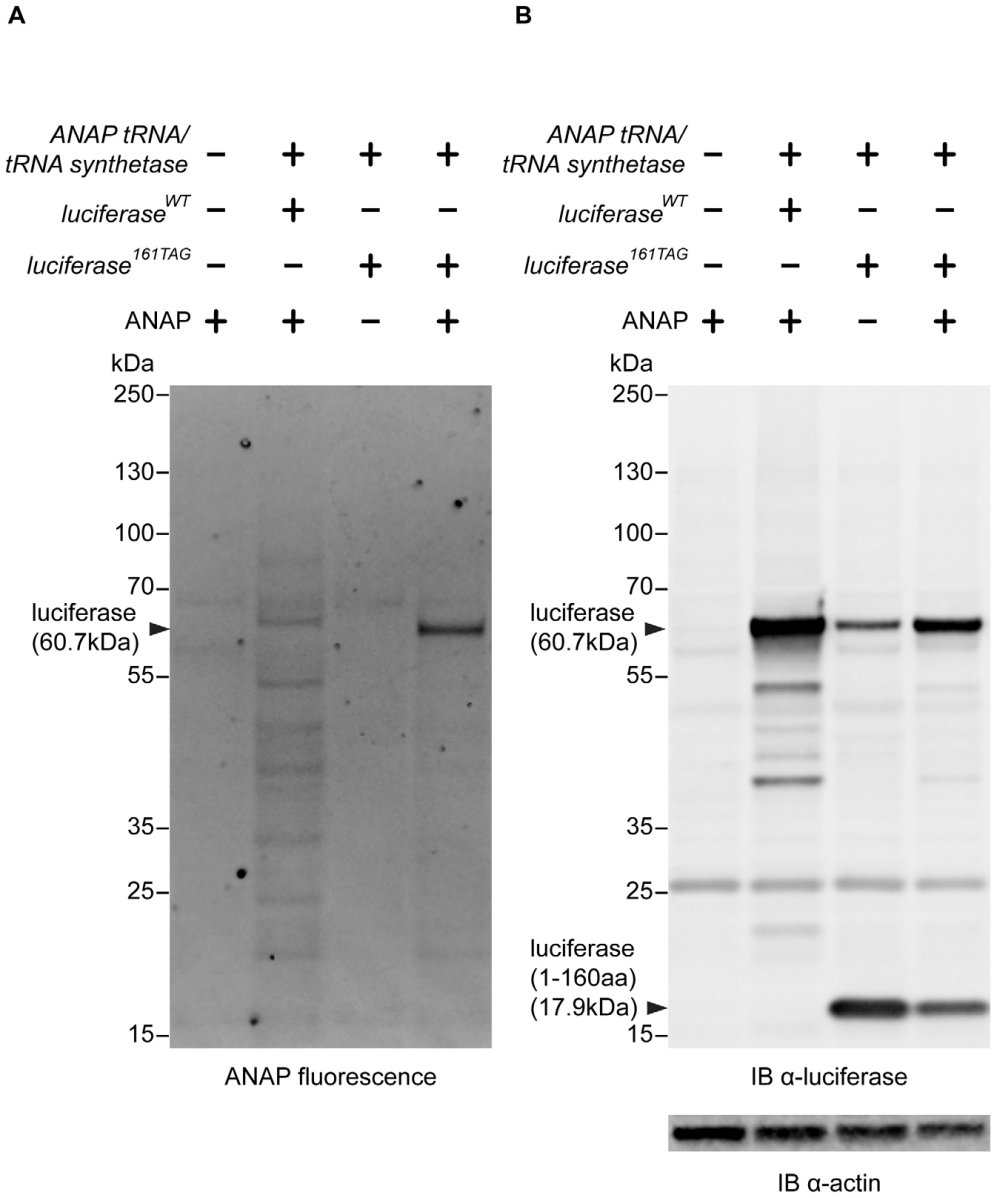
Specific incorporation of ANAP in Luc F161ANAP. Specific incorporation of ANAP in Luc F161ANAP expressed in OT56 [*PSI* ^+^] cells analyzed by SDS-PAGE of total protein extracts and scanning of the gel for ANAP fluorescence (excitation wavelength 365 nm, emission wavelength 510 nm). (A) Immunoblotting analysis of Luc F161ANAP expression levels using luciferase specific antiserum. Immunodetection of α-Actin served as a loading control.

About 17% of yeast genes (∼1000 genes) are terminated by a TAG codon [14]. This raises the possibility of non-specific incorporation of ANAP at the C-terminus of such yeast proteins, especially under reduced soluble Sup35 levels. To address this issue we analyzed ANAP fluorescence of total protein lysates of cells grown in the presence of ANAP by SDS-PAGE and fluorescence scanning of gels (Figure 2A). As expected, the appearance of fluorescent protein depended on the presence of ANAP and the plasmid encoded tRNA/tRNA-synthetase pair. In the absence of the plasmid encoding for Luc F161ANAP we detected background fluorescence of a few proteins which most likely were labeled upon suppression of their natural amber stop codon thereby producing a C-terminally extended protein. Importantly, in cells expressing Luc F161ANAP, the non-specific labeling of these yeast proteins was strongly suppressed and instead we almost exclusively detected Luc F161ANAP, as verified by the apparent molecular weight in SDS-PAGE and western blot analysis (Figure 2B). We also note that in the absence of ANAP, cells expressing the ANAP incorporation system partially suppress the TAG in luciferase mutant genes by incorporation of another amino acid (Figure 2B, lane 3) but this suppression is much weaker as compared to the suppression by ANAP incorporation. In agreement with this finding cells expressing Luc F161ANAP show strongly increased fluorescence compared to cells expressing wild type luciferase (Figure S2). We conclude that our experimental system facilitates the specific incorporation of ANAP in Luc F161ANAP with negligible background incorporation of ANAP into endogenous yeast proteins.

### Microscopic detection of heat induced luciferase misfolding

**Figure 3:**
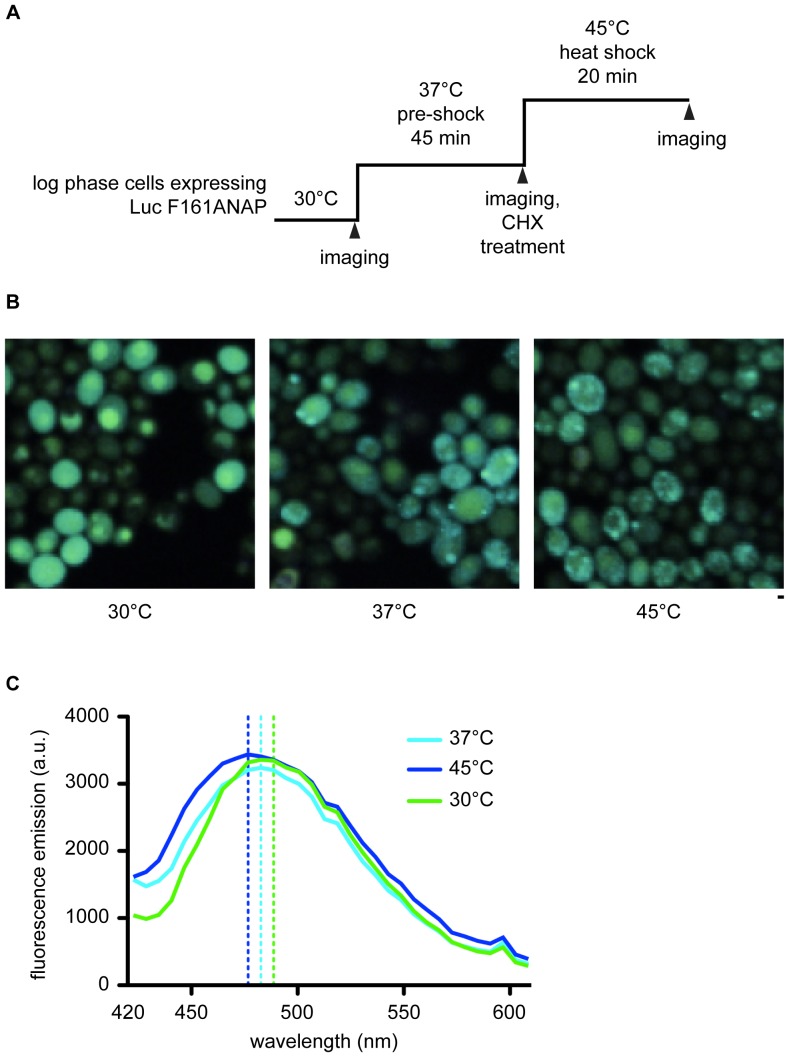
ANAP fluorescence emission changes report on heat-induced misfolding and aggregation of Luc F161ANAP *in vivo*. Schematic overview of the heat shock regime for analyzing Luc F161ANAP misfolding and aggregation. Cells were grown at 30°C to logarithmic phase and exposed to mild heat-shock (37°C) for 45 min. Cycloheximide (CHX) was added to arrest protein synthesis and cells were shifted to 45°C for 20 min. Samples were taken for microscopic imaging of ANAP fluorescence as indicated. (A) Microscopic analysis of yeast cells expressing Luc F161ANAP after incubation at different temperatures as indicated in (A). Heat shock changes the localization and the fluorescence emission color of Luc F161ANAP. Shown are overlays of all 32 channels. Bar 1 µm. (B) Fluorescence emission spectrum of Luc F161ANAP in cells exposed to 30°C, 37°C or 45°C. The fluorescence emission spectra of the cytosol of 50 cells (30°C and 37°C) or 50 aggregate foci (45°C) were analyzed and the average fluorescence measured at the different wavelengths was plotted. The color-coded dashed lines align to the wavelength of maximum emission. a.u.  =  arbitrary units.

We next performed heat-shock experiments of cells expressing Luc F161ANAP and analyzed whether unfolding of luciferase causes a shift of ANAP fluorescence that can be visualized by microscopy. To capture the ANAP fluorescence emission spectrum, images were acquired using a 32-channel spectra detector at a resolution of 6 nm per channel covering a wavelength range of 420 to 610 nm and the resulting spectra were overlaid. The prodan fluorophore in ANAP is environmentally sensitive and shows a green to blue shift of the emission maximum when shifted from a hydrophilic to hydrophobic surrounding. In Luc F161ANAP, ANAP is incorporated into a solvent exposed loop placing the prodan fluorophore in a rather hydrophilic environment. Accordingly, cells grown at 30°C show green fluorescence that is homogeneously distributed in the cytoplasm, which suggests that Luc F161ANAP is soluble and natively folded (Figure 3A, B). Exposing the cells to a mild heat shock at 37°C already reduced the green fluorescence distributed in the cytoplasm and caused the appearance of diffuse blue fluorescence and bright blue foci. These phenomenon was further increased upon shift to 45°C. Since the synthesis of new protein was blocked by cycloheximide before heat-shock, these foci must originate from folded luciferase that misfolded and aggregated upon heat treatment. The shift from green to blue fluorescence suggested that denaturation and aggregation of Luc F161ANAP increased the hydrophobicity of the local environment of prodan which resulted in a blue shift of the emitted light. Analysis of the fluorescence emission spectra of the cytoplasm of 50 cells grown at 30 or 37°C and 50 blue foci from heat-shocked cells (45°C) by confocal microscopy revealed a shift of the emission peak from 489 nm (30°C) to 483 nm (37°C) to 477 nm upon severe heat shock (Figure 3C). Together these data indicated a direct correlation of the blue shift of the emitted light with increased average hydrophobicity of the prodan environment on position F161 caused by luciferase unfolding and aggregation.

### Quantifications of hydrophobicity changes in individual cells by flow cytometry

**Figure 4:**
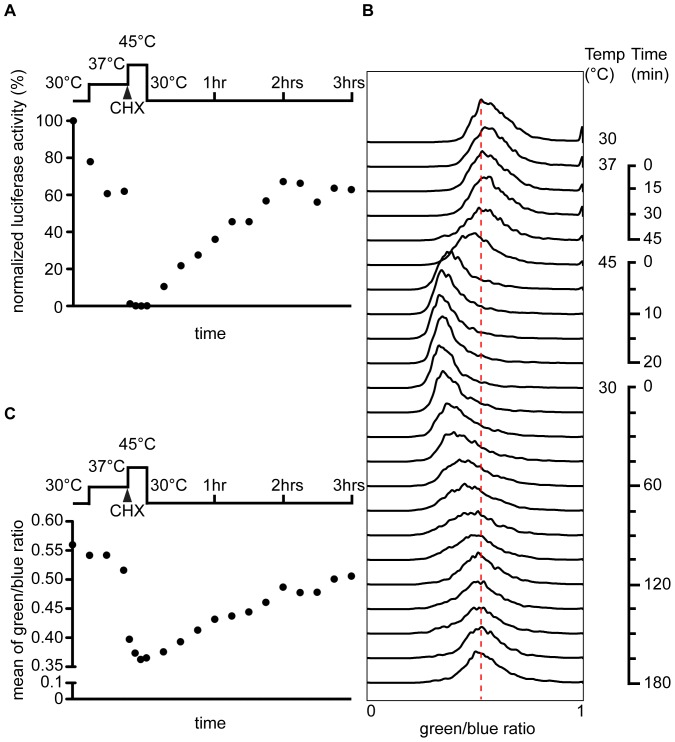
Changes in the folding state of Luc F161ANAP detected by flow cytometry. Normalized luciferase activity of cells expressing Luc F161ANAP before heat-shock (30°C) and during pre-shock (37°C), heat shock (45°C) and recovery (30°C). The activity value measured before the temperature was elevated from 30°C to 37°C was set to 100%. CHX, addition of cycloheximide for blocking protein synthesis. (A) Histogram of the ratio of green (510 nm) to blue (450 nm) fluorescence of yeast cells expressing Luc F161ANAP at different temperatures and time-points as described in (A). The red dash line aligns to the peak position before heat-induced unfolding of Luc F161ANAP. (B) Mean of the ratio of green (510 nm) to blue (450 nm) fluorescence light emission of about 5000 yeast cells each analyzed at indicated temperatures and time-points as described in (A).

We also explored the possibility of analyzing conformational changes of luciferase by detecting changes in the fluorescence emission profile of cells using flow cytometry. Cells in logarithmic phase were shifted from media containing raffinose to media containing galactose and ANAP to induce Luc F161ANAP synthesis. After seven hours of induction cells were exposed first to a mild heat-shock at 37°C for 45 min followed by cycloheximide (CHX) treatment and severe heat-shock at 45°C for 20 min. Subsequently, cells were shifted back to 30°C for 3 hours to allow protein disaggregation and refolding. Throughout the experiment, the folding state of Luc F161ANAP was analyzed by activity measurements (Figure 4A) and by flow cytometry detecting the emission of blue (450 nm) and green light (510 nm) (Figure 4B). Before heat treatment of cells luciferase was folded and active. Elevating the temperature to 37°C reduced the luciferase activity (Figure 4A). This partial inactivation coincided with a shift of the ratio of green to blue light emitted by the prodan moiety (Figure 4B, C). This effect is strongly exacerbated with a further temperature upshift to 45°C. This temperature led to complete loss of luciferase activity and a drop of the ratio of green to blue light emission (Figure 4B, C and Figure S3). Shifting the cells back to 30°C led to recovery of about 70% of the luciferase activity and a change of the ratio of green to blue fluorescence from 0.36 to ∼0.5 during the 3 hours of recovery (Figure 4C). This finding suggested that fluorescence emission changes directly reflect the chaperone-mediated refolding of denatured luciferase.

### Fluorescence emission changes indicating the refolding of heat-denatured Luc F161ANAP depend on the chaperone Hsp104

To support the conclusion that the measured fluorescence emission changes are caused by F161ANAP refolding, we repeated the refolding experiment using a yeast strain that due to absence of the chaperone Hsp104 is incapable to disaggregate and refold the aggregated luciferase. Since *hsp104Δ* strains cannot maintain the [*PSI* ^+^] state we constructed a new strain (W303_Ub*35*) in which the level of Sup35 is artificially reduced by creating an N-degron on Sup35 [15]. This manipulation reduced the Sup35 levels to about 50% and elevated the level of Luc F161ANAP about twofold compared to wild-type W303 (Figure S4A, B). The dependence of Luc F161ANAP expression on level of functional Sup35 is also visible in microscopy images taken with identical settings for W303 or W303_UB*35* cells (Figure S4C).

**Figure 5:**
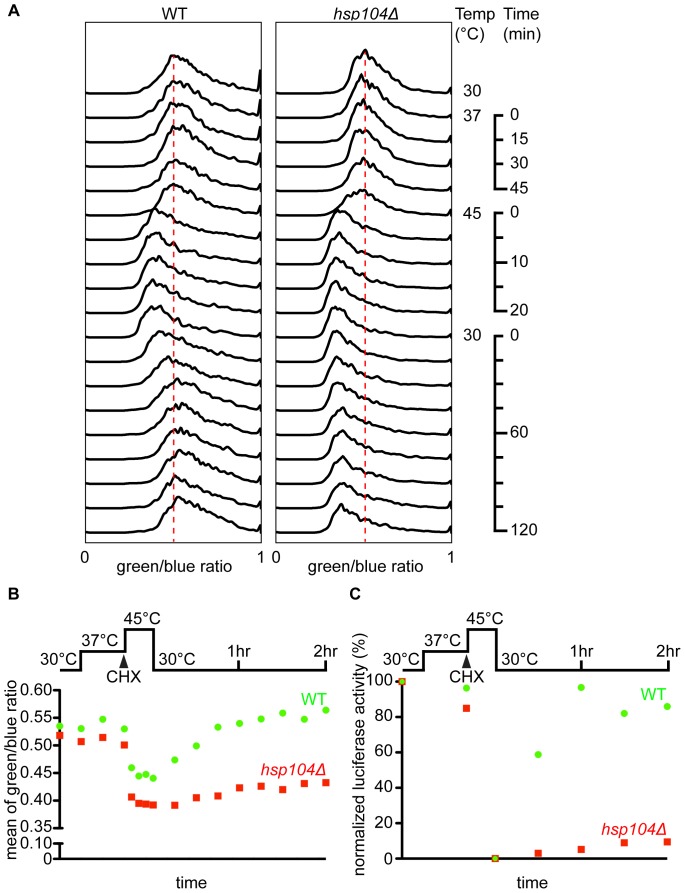
Hsp104-dependent refolding of Luc F161ANAP analyzed by flow cytometry. Histogram of the ratio of green (510 nm) to blue (450 nm) fluorescence of wild-type Sup35-destabilized W303 and W303*hsp104Δ* cells expressing Luc F161ANAP. After pre-shock and heat shock, cells were allowed to recover for 2 hours at 30°C. Flow cytometry was performed every 15, 5, and 15 minutes during pre-shock, heat shock and recovery, respectively. Red dash lines align to the peak position before heat shock. (A) Quantification of Luc F161ANAP refolding shown as mean of the ratio of green (510 nm) to blue (450 nm) fluorescence. Green circles represent fluorescence ratios from wild-type cells, and the red squares represent fluorescence ratios from *hsp104Δ* cells. (B) Normalized luciferase activity of wild-type and *hsp104Δ* cells expressing Luc F161ANAP before heat shock, during heat shock and during recovery. The luciferase activity measured before temperature upshift from 30°C to 37°C was set to 100%. Green circles indicate the relative luciferase activity of wild-type cells, and the red squares of *hsp104Δ* cells.

Strain W303_Ub*35* and the isogenic mutant lacking *hsp104* were used to analyze the disaggregation and refolding of Luc F161ANAP by flow cytometry (Figure 5A). W303_Ub*35* cells containing Hsp104 exhibited a comparable change in the ratio of green to blue light emission during heat shock and recovery as observed earlier in OT56 [*PSI* ^+^] (Figures 4C and 5B). Cells lacking Hsp104 showed slightly lower ratio of green to blue fluorescence at 30 and 37°C, which suggests minor folding deficiencies of Luc F161ANAP. Heat-induced unfolding of luciferase at 45°C changed the ratio of green to blue light emission to about 0.44 and 0.39 for the wild type and *hsp104Δ* mutant cells, respectively. Importantly, during recovery at 30°C changes in ratio of green to blue ANAP fluorescence (Figure 5A, B) as well as luciferase activity (Figure 5C) occurred only in wild-type cells but not *hsp104Δ* cells. This finding is in excellent agreement with the fact that Hsp104 is strictly required for protein disaggregation and strongly supports our assumption that changes in the emission spectra of ANAP can be directly used for measuring unfolding and refolding of proteins in living cells.

## Discussion

Incorporation of an unnatural amino acid *in vivo* by introducing an orthogonal tRNA/tRNA synthetase to suppress the TAG amber stop codon is a powerful tool for generating proteins with new functional groups in a site-specific manner. The number of systems, the range of unnatural amino acids with new chemical and physical properties and the range of organisms that are tractable are rapidly growing. Examples are amino acids with side chains that are photo- or chemically reactive, photoisomerizable, photocaged, facilitate posttranslational modifications, allow metal binding or contain fluorophores [7]. Adding such properties to specific positions in proteins offers a wide range of new possibilities for studying structure and function of proteins *in vivo* and *in vitro*.

To date, fluorescent labeling of proteins *in vivo* has been used mainly for two purposes, either for localizing the labeled protein *in vivo* or for *in vitro* analysis of the purified fluorescent proteins. Our proof-of principle study extends the potential use of site-specific fluorescent labeling of proteins *in vivo* demonstrating that ANAP incorporation and the analysis of fluorescence emission spectra facilitates the study of unfolding, aggregation and refolding of a protein in the context of a living cell. We show that denaturation-induced misfolding is detectable by spectral analysis of ANAP fluorescence of individual yeast cells with non-invasive confocal microscopy or flow cytometry. Importantly, detected shifts of the emission maximum of ANAP directly correlate with a loss of luciferase activity and the formation of large protein aggregates in light microscopy, which implies that fluorescence changes directly report on conformational changes in luciferase.

A critical prerequisite for studying the folding of a specific protein *in vivo* is that background ANAP labeling of authentic proteins is low. In fact we find this is the case. In the absence of the plasmid encoding for Luc F161ANAP, only a few rather dim protein bands are visible upon scanning of SDS gels for ANAP fluorescence where entire cell lysates have been analyzed and this unspecific incorporation is further reduced if Luc F161ANAP is expressed. Furthermore, microscopic analysis of cells grown in the presence of ANAP reveals very weak background fluorescence (Figure S2).

In Luc F161ANAP, the fluorescent probe is located on a surface-exposed loop of the protein. Compared to a positioning of ANAP in the hydrophobic core, the exchange of a surface exposed amino acid has the advantage that the functional integrity of the mutant protein is less likely to be impaired. The disadvantage is that the prodan fluorophore has a rather polar environment already in the folded protein and therefore unfolding is less likely to significantly increase this polarity. In case of luciferase, unfolding even caused a blue shift of fluorescence, which indicates a more hydrophobic environment of ANAP, most likely upon aggregation of the misfolded protein.

Different experimental systems are currently employed for studying protein folding and unfolding *in vivo*. This involves activity measurements, biochemical analysis of the protein solubility following cell lysis, labeling of protein aggregates and amyloids with specific dyes such as Congo red or studying the distribution of fluorescently labeled proteins within cells by light microscopy. Each approach however has specific limitations. Activity measurements are limited to a small number of proteins for which such assays are available. Solubility assays rely on enrichment procedures that distinguish native from aggregated protein fractions based on their sedimentation during low-speed centrifugation and thus cannot be used in living cells. Staining aggregates or amyloids with Congo red has limited specificity and sensitivity. Genetic fusion of fluorescent protein adds a large additional domain to the protein studied, which can affect structure, localization, activity, and interaction properties of the tagged proteins. These constraints are avoided by the site-specific incorporation of ANAP into a protein of interest. Despite of the necessity to co-express the tRNA/tRNA synthetase pair, to reduce translation termination for improving ANAP incorporation, and to identify an ANAP incorporation site that does not perturb protein structure, ANAP fluorescence measurements may provide a superior tool for time-resolved *in vivo* measurements of misfolding, chaperone-mediated refolding and subcellular localization of distinct proteins in living cells. We also notice that the ANAP fluorescence emission system is more sensitive than the alternative methods. Already upon shift from 30 to 37°C and before formation of large aggregates is observed, we detect a small but significant blue shift in fluorescence emission spectra (Figure 3C), suggesting partial unfolding of luciferase. Importantly, this shift directly correlates with a partial reduction in the luciferase activity. Finally ANAP fluorescence labeling is the only method that has the potential to report on protein misfolding that does not coincide with aggregation.

Studying conformational changes of proteins in living cells is a major challenge and of great interest for basic and applied research. Misfolding and aggregation of distinct proteins are implicated in a wide variety of fatal human diseases, including Alzheimers disease, Parkinson disease, type 2 diabetes, prions disease and familial lateral sclerosis (fALS)[4], [5]. Since the ANAP incorporation system is now available for mammalian cells [10], the experimental setup described here for yeast now represents a potential new tool for studying such protein conformational disorders in the context of live mammalian cells. The high sensitivity of ANAP fluorescence spectra analysis and the possibility to employ flow cytometry for high-throughput analysis of individual cells may allow for studying different stages of disease formation upon protein conformational changes and aggregation, including the early formation of potentially harmful protein oligomers as well as the formation of amyloid deposits in a wide variety of pathological conditions of living cells.

## Materials and Methods

### ANAP synthesis and in vivo incorporation procedure

The unnatural amino acid, 3-(6-acetylnaphthalen-2-ylamino)-2-aminopropanoic acid (ANAP), was synthesized as described [9]. The structure of the synthesis compound was confirmed by ^1^H nuclear magnetic resonance (NMR) spectroscopy and mass spectrometry. High-performance liquid chromatography revealed that the synthesis compound reached 97.3% purity. The plasmid encoding the evolved tRNA/tRNA synthetase pair for ANAP incorporation, pSNR-ProRS, was obtained from the lab of Prof. Peter G. Schultz (The Scripps Research Institute, La Jolla, CA 92037, USA). For *in vivo* incorporation, ANAP was dissolved in 0.1 M NaOH and mixed with synthetic drop out medium to a final concentration of 0.4 mM, followed by filter sterilization using a 0.2 µm filter. For experiments involving live cell fluorescence detection, a low fluorescence synthetic drop out medium lacking riboflavin and folic acid was used (YNB-Folic Acid-Riboflavin, Sunrise Science products, San Diego, CA, USA).

### Construction of plasmids facilitating synthesis of ANAP-labeled luciferase

Plasmid pLuzi expressing thermolabile luciferase was constructed using pRS425GalI [16] containing a galactose inducible promoter as plasmid backbone and plasmid pRS306-PGpd Thorn-mCherry-luci [17] for PCR amplification of the gene encoding luciferase. The positions for ANAP incorporation were selected based on the X-ray crystallographic structure of luciferase (PDB accession number: 1LCI, [18]). Eight hydrophobic amino acids that are either exposed or buried in the core of native luciferase were selected: Y15, F127, F161, F247, F273, T340, W417, F465. The amber TAG codon for ANAP incorporation was introduced by use of the Quikchange site-directed mutagenesis protocol (Stratagene). DNA oligonucleotide sequences for constructing Luc F161ANAP were as follows:

F-QC-luci-161: 5'-aacggattaccagggaTAGcagtcgatgtacacgt-3'

R-QC-luci-161: 5'-acgtgtacatcgactgCTAtccctggtaatccgtt-3'

DNA oligonucleotides for constructing TAG luciferase mutant genes are described in Table S2 in File S1.

### Determination of specific luciferase activity

Luciferase activity of cell synthesizing wild-type luciferase or ANAP-labeled luciferase mutants was measured *in vivo* by use of the Promega Dual-luciferase reporter assay system. The specific luciferase activity was obtained by normalizing the luciferase activity to absolute luciferase levels detected by Western blot analysis and quantification by use of Image Reader software.

### Analysis of ANAP incorporation specificity

ANAP incorporation specificity was analyzed by separation of cell lysates with standard SDS-PAGE followed by scanning the gel for ANAP fluorescence emission by use of the Fuji LAS-4000 instrument equipped with 365 nm excitation and 510 nm emission filters.

### Microscopy analysis of in vivo ANAP fluorescence emission spectra

Yeast cells were grown to logarithmic phase (OD_600_≈0.5) in synthetic dropout media containing 2% raffinose. Cells were pelleted by low-speed centrifugation at 1000 g for 5 min, resuspended in low fluorescence synthetic drop out medium (YNB-Folic Acid-Riboflavin medium) supplemented with 2% galactose and 0.4 mM ANAP and growth was continued for 7 hours in the dark. Cells were washed 4 times with phosphate buffered saline plus 2% glucose to remove residual ANAP. Microscopy images were acquired with a Nikon A1R confocal microscope equipped with a Nikon Plan Apo 60× NA 1.40 oil immersion objective lens and a 32-channel spectrum detector. ANAP was excited at 405 nm, and the emitted light was detected with a wavelength resolution of 6 nm per channel to monitor fluorescence emission between 420 and 610 nm. After image acquisition, regions of interest within cells were selected and fluorescence emission spectra were analyzed.

### Analysis of ANAP fluorescence emission spectrum in vivo by flow cytometry

Yeast cells were grown and washed as described before for fluorescence emission spectra analysis by microscopy. Washed yeast cells were resuspended in low fluorescence synthetic drop out medium and analyzed by use of the FACScanto instrument (Becton-Dickinson). ANAP was excited with a 405 nm laser, and fluorescence emission was measured using a green channel (filter: 510/50) and a blue channel (filter: 450/50). To avoid background fluorescence of cells, the voltage of the photomultiplier was adjusted by use of cells grown in the absence of ANAP. The relative fluorescence intensity (pulse area) was adjusted to 100 units. A total of 30,000 events were collected. Yeast cell expressing Luc F161ANAP with green and blue fluorescence intensity exceeding 500 units were defined as a gated subpopulation with elevated Luc F161ANAP levels for further analysis. The fluorescence intensity ratio of green to blue channel was plotted on a histogram. After collecting the initial data set for cells grown at 30°C, the temperature was raised to 37 °C for a 45-min pre-shock. Subsequently, cycloheximide was added to a final concentration of 0.1 mg/ml for instant inhibition of protein synthesis, and the temperature was raised to 45°C for 20 min. The cells were then cooled to 30°C for recovery as indicated. During pre-shock, heat shock and recovery, samples were taken for analysis of the fluorescence emission using flow cytometry and for luciferase activity measurements.

### Destabilized Sup35 W303 strain

The yeast Sup35 in strains W303 and W303 *hsp104Δ* was destabilized by replacing the *sup35* locus with a cassette encoding N-terminal ubiquitin fused to *sup35* that contains an arginine instead of the N-terminal methionine. The genetic construct integrated in the genome consisted of a 55 bp 5′-UTR upstream of the *sup35* start codon, followed by sequences encoding ubiquitin, full-length *sup35* with N-terminal methionine replaced with arginine, an HA tag, a clonNat antibiotic selection marker derived from plasmid pFA6a-natNT2, and a 57 bp sequence stretch derived from the 3′-UTR downstream of the *sup35* termination codon. For construction of this fragment individual sequence stretches were amplified and fused into the full-length cassette by overlapping PCR with the primers described in Table S3 in File S1.

## Supporting Information

Figure S1 **Reduced translation termination efficiency in [** ***PSI*** **^+^] cells improves the efficiency of amber codon suppression by ANAP incorporation.** ANAP incorporation is elevated in [*PSI* ^+^] cells. Immunoblot (IB) using luciferase antisera for detecting luciferase and Luc F161ANAP in isogenic [*PSI* ^+^] and [*psi* ^−^] yeast cells. IB with antisera raised against actin served as a loading control. (A) [*PSI* ^+^] dependent ANAP incorporation in Luc F161ANAP analyzed by fluorescence microscopy. Identical microscope settings were used to compare the Luc F161ANAP fluorescence in [*PSI* ^+^] and [*psi* ^−^] cells. Shown are overlays of all 32 channels. Bar, 1 µm.
(TIF)

Figure S2 **Unspecific incorporation of ANAP in endogenous proteins analyzed by fluorescence microscopy.** ANAP fluorescence intensity of OT56 [*PSI* ^+^] cells lacking the ANAP incorporation system (left), cells expressing the ANAP incorporation system and wild-type luciferase (middle), or cells expressing both Luc F161ANAP and the ANAP incorporation system (right). Shown are overlays of all 32 channels. Bar, 1 µm.
(TIF)

Figure S3 **Temperature-dependent ANAP fluorescence profile changes in OT56 [** ***PSI*** **^+^] cells expressing Luc F161ANAP detected by flow cytometry.** Histogram of the ratio of green (510 nm) to blue (450 nm) fluorescence of yeast cells expressing Luc F161ANAP before (30°C) and after (45°C) heat shock.
(TIF)

Figure S4 **Reduced Sup35 levels in W303_Ub** ***35*** **increase the efficiency of amber codon suppression by ANAP incorporation.** Immunodetection of Sup35 shows the *in vivo* concentration of Sup35 in W303_Ub*35* is reduced by N-terminal fusion of ubiquitin. Lower panel: Sup35 levels were determined by quantifying western blots using Fujifilm LAS 4000 equipment and Image Reader software. (A) Immunoblotting demonstrates that ANAP incorporation in Luc F161ANAP is improved by destabilization of Sup35 in strain W303_Ub*35*. Lower panel: Luciferase levels were determined by quantifying western blots using Fujifilm LAS 4000 equipment and Image Reader software. (B) Increased ANAP incorporation efficiency by destabilizing Sup35 in W303_Ub*35* analyzed by fluorescence microscopy. Identical microscope settings were used to compare the Luc F161ANAP yield in both strains. Shown are overlays of all 32 channels. Bar, 1 µm.
(TIF)

File S1 **This contains Table S1, S2, and S3.** Table S1 provides additional information on additional luciferase mutants constructed and analyzed. Table S2 provides the sequences of the DNA oligonucleotides used for luciferase mutant construction and Table S3 provides the sequences of the DNA oligonucleotide used for constructing W303_Ub*35* encoding destabilized Sup35.
(DOCX)

## References

[1] Dobson CM, Ellis RJ (1998) Protein folding and misfolding inside and outside the cell. EMBO J 17: 5251–5254. 9736604 10.1093/emboj/17.18.5251 PMC1170852

[2] Tyedmers J, Mogk A, Bukau B (2010) Cellular strategies for controlling protein aggregation. Nat Rev Mol Cell Biol 11: 777–788. 20944667 10.1038/nrm2993

[3] Kim YE, Hipp MS, Bracher A, Hayer-Hartl M, Hartl FU (2013) Molecular chaperone functions in protein folding and proteostasis. Annu Rev Biochem 82: 323–355. 23746257 10.1146/annurev-biochem-060208-092442

[4] Dobson CM (1999) Protein misfolding, evolution and disease. TiBS 24: 329–332. 10470028 10.1016/s0968-0004(99)01445-0

[5] Soto C (2003) Unfolding the role of protein misfolding in neurodegenerative diseases. Nat Rev Neurosci 4: 49–60. 12511861 10.1038/nrn1007

[6] Landgraf D, Okumus B, Chien P, Baker TA, Paulsson J (2012) Segregation of molecules at cell division reveals native protein localization. Nat Methods 9: 480–482. 22484850 10.1038/nmeth.1955 PMC3779060

[7] Liu CC, Schultz PG (2010) Adding new chemistries to the genetic code. Annu Rev Biochem 79: 413–444. 20307192 10.1146/annurev.biochem.052308.105824

[8] Weber G, Farris FJ (1979) Synthesis and spectral properties of a hydrophobic fluorescent probe: 6-propionyl-2-(dimethylamino)naphthalene. Biochemistry 18: 3075–3078. 465454 10.1021/bi00581a025

[9] Lee HS, Guo J, Lemke EA, Dimla RD, Schultz PG (2009) Genetic incorporation of a small, environmentally sensitive, fluorescent probe into proteins in Saccharomyces cerevisiae. J Am Chem Soc 131: 12921–12923. 19702307 10.1021/ja904896s PMC2873857

[10] Chatterjee A, Guo J, Lee HS, Schultz PG (2013) A genetically encoded fluorescent probe in mammalian cells. J Am Chem Soc 135: 12540–12543. 23924161 10.1021/ja4059553 PMC3783214

[11] Glover JR, Lindquist S (1998) Hsp104, Hsp70, and Hsp40: A novel chaperone system that rescues previously aggregated proteins. Cell 94: 73–82. 9674429 10.1016/s0092-8674(00)81223-4

[12] Stansfield I, Jones KM, Kushnirov VV, Dagkesamanskaya AR, Poznyakovski AI, et al (1995) The products of the SUP45 (eRF1) and SUP35 genes interact to mediate translation termination in Saccharomyces cerevisiae. EMBO J 14: 4365–4373. 7556078 10.1002/j.1460-2075.1995.tb00111.x PMC394521

[13] Sadlish H, Rampelt H, Shorter J, Wegrzyn RD, Andreasson C, et al (2008) Hsp110 chaperones regulate prion formation and propagation in S. cerevisiae by two discrete activities. PLoS ONE 3: e1763. 18335038 10.1371/journal.pone.0001763 PMC2258148

[14] Nakamura Y, Gojobori T, Ikemura T (2000) Codon usage tabulated from international DNA sequence databases: status for the year 2000. Nucleic Acids Res 28: 292. 10592250 10.1093/nar/28.1.292 PMC102460

[15] Bachmair A, Finley D, Varshavsky A (1986) In vivo half-life of a protein is a function of its amino-terminal residue. Science 234: 179–186. 3018930 10.1126/science.3018930

[16] Mumberg D, Muller R, Funk M (1994) Regulatable promoters of Saccharomyces cerevisiae: comparison of transcriptional activity and their use for heterologous expression. Nucleic Acids Res 22: 5767–5768. 7838736 10.1093/nar/22.25.5767 PMC310147

[17] Specht S, Miller SB, Mogk A, Bukau B (2011) Hsp42 is required for sequestration of protein aggregates into deposition sites in Saccharomyces cerevisiae. J Cell Biol 195: 617–629. 22065637 10.1083/jcb.201106037 PMC3257523

[18] Conti E, Franks NP, Brick P (1996) Crystal structure of firefly luciferase throws light on a superfamily of adenylate-forming enzymes. Structure 4: 287–298. 8805533 10.1016/s0969-2126(96)00033-0

