# Supplementary figures and images for: Monitoring Protein Misfolding by Site-Specific Labeling of Proteins In Vivo
Source: PLoS One. 2014 Jun 10;9(6):e99395. doi: 10.1371/journal.pone.0099395 (PMC4051779; doi:10.1371/journal.pone.0099395)

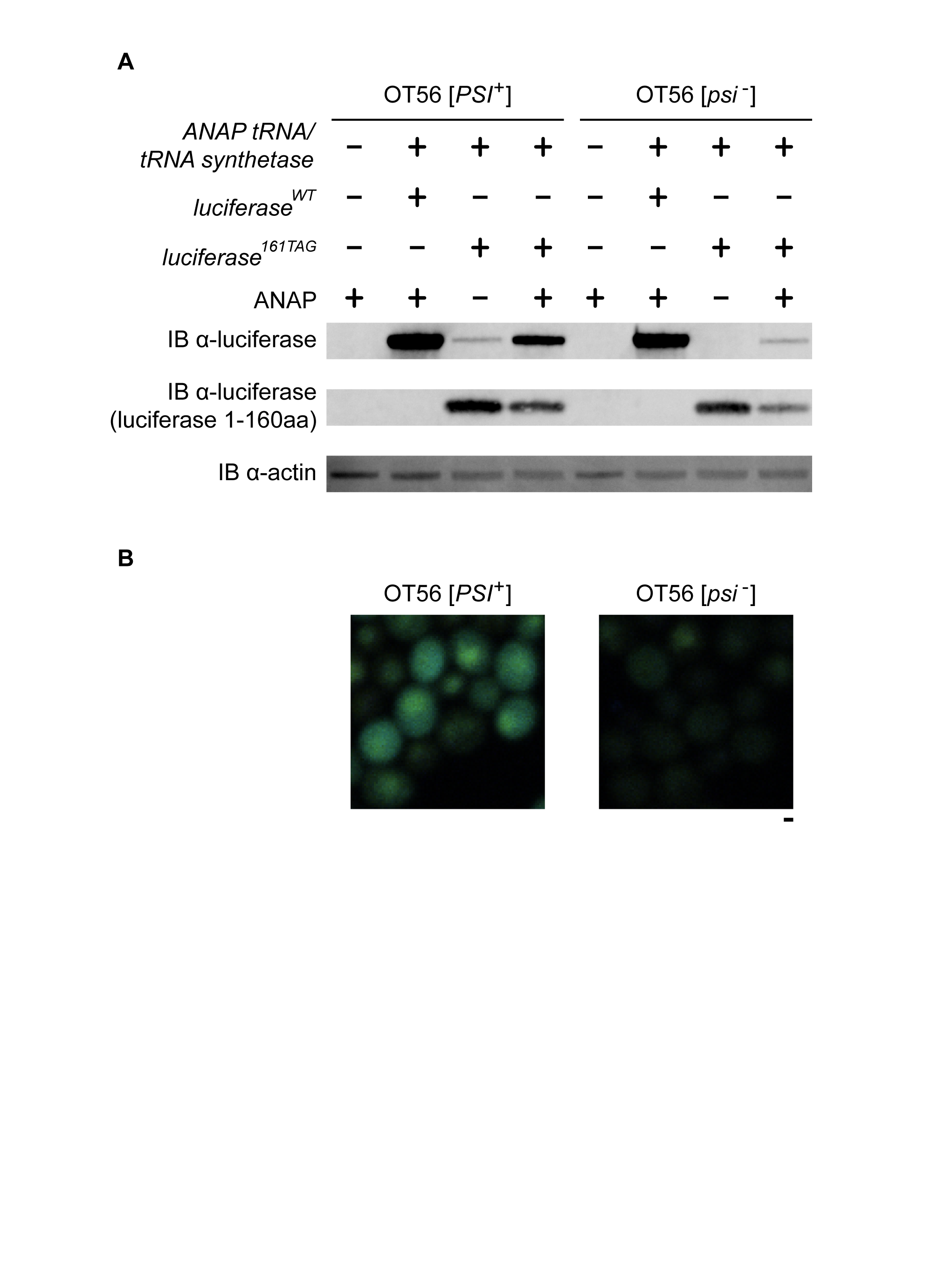

Supplement: Figure S1 — Reduced translation termination efficiency in [ PSI +] cells improves the efficiency of amber codon suppression by ANAP incorporation. ANAP incorporation is elevated in [PSI +] cells. Immunoblot (IB) using luciferase antisera for detecting luciferase and Luc F161ANAP in isogenic [PSI +] and [psi −] yeast cells. IB with antisera raised against actin served as a loading control. (A) [PSI +] dependent ANAP incorporation in Luc F161ANAP analyzed by fluorescence microscopy. Identical microscope settings were used to compare the Luc F161ANAP fluorescence in [PSI +] and [psi −] cells. Shown are overlays of all 32 channels. Bar, 1 µm. (TIF) [file pone.0099395.s001.tif]

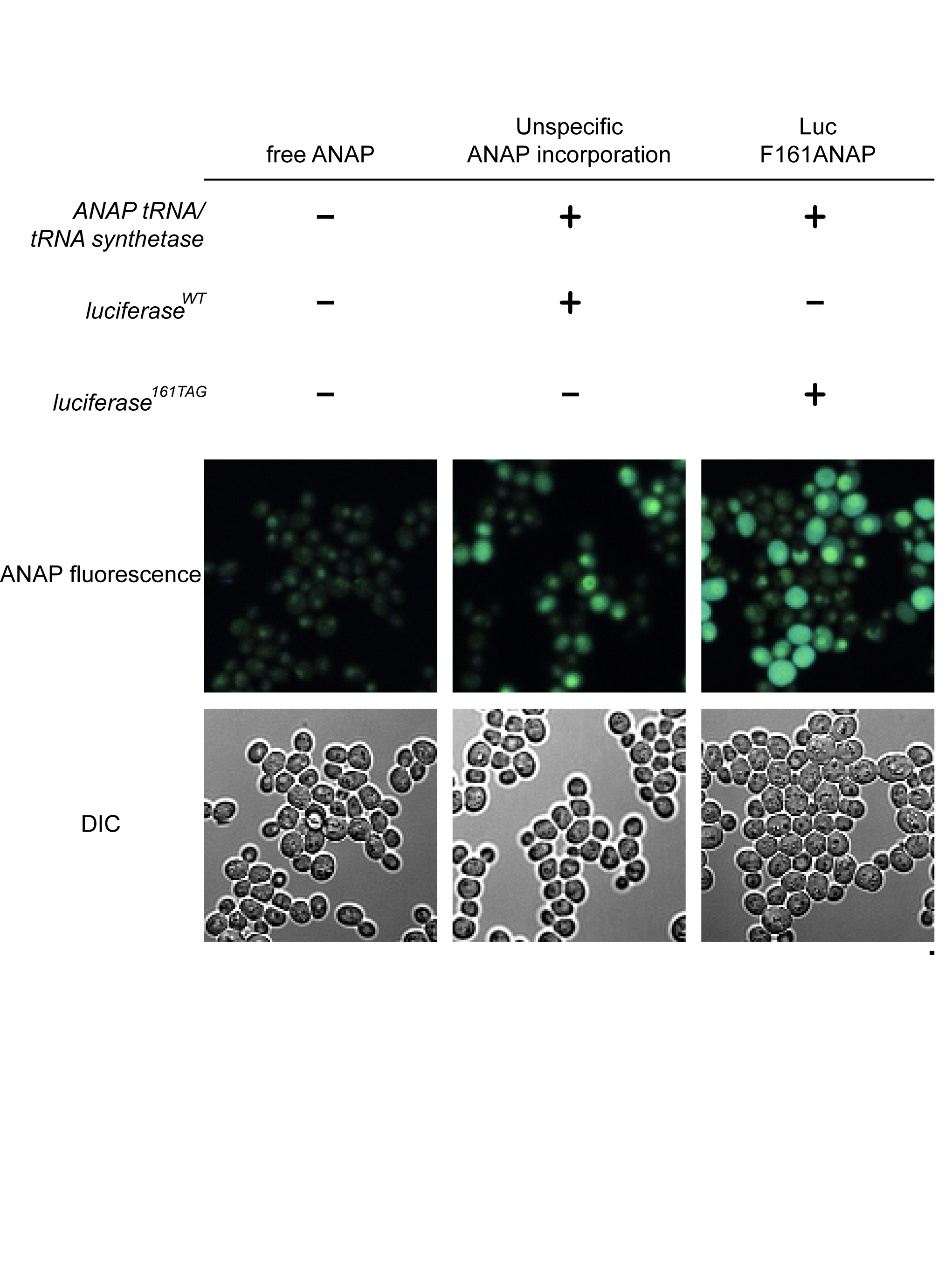

Supplement: Figure S2 — Unspecific incorporation of ANAP in endogenous proteins analyzed by fluorescence microscopy. ANAP fluorescence intensity of OT56 [PSI +] cells lacking the ANAP incorporation system (left), cells expressing the ANAP incorporation system and wild-type luciferase (middle), or cells expressing both Luc F161ANAP and the ANAP incorporation system (right). Shown are overlays of all 32 channels. Bar, 1 µm. (TIF) [file pone.0099395.s002.tif]

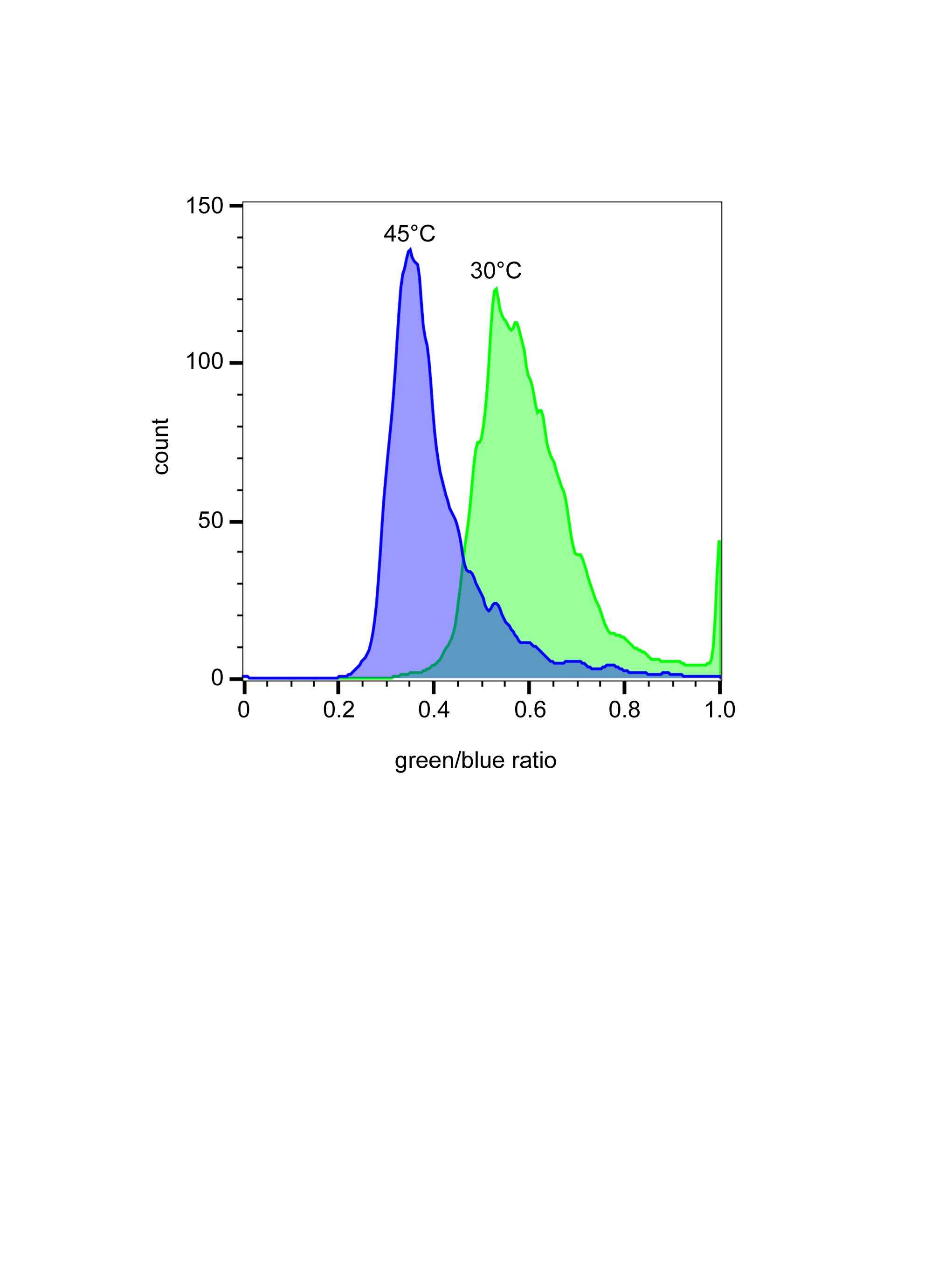

Supplement: Figure S3 — Temperature-dependent ANAP fluorescence profile changes in OT56 [ PSI +] cells expressing Luc F161ANAP detected by flow cytometry. Histogram of the ratio of green (510 nm) to blue (450 nm) fluorescence of yeast cells expressing Luc F161ANAP before (30°C) and after (45°C) heat shock. (TIF) [file pone.0099395.s003.tif]

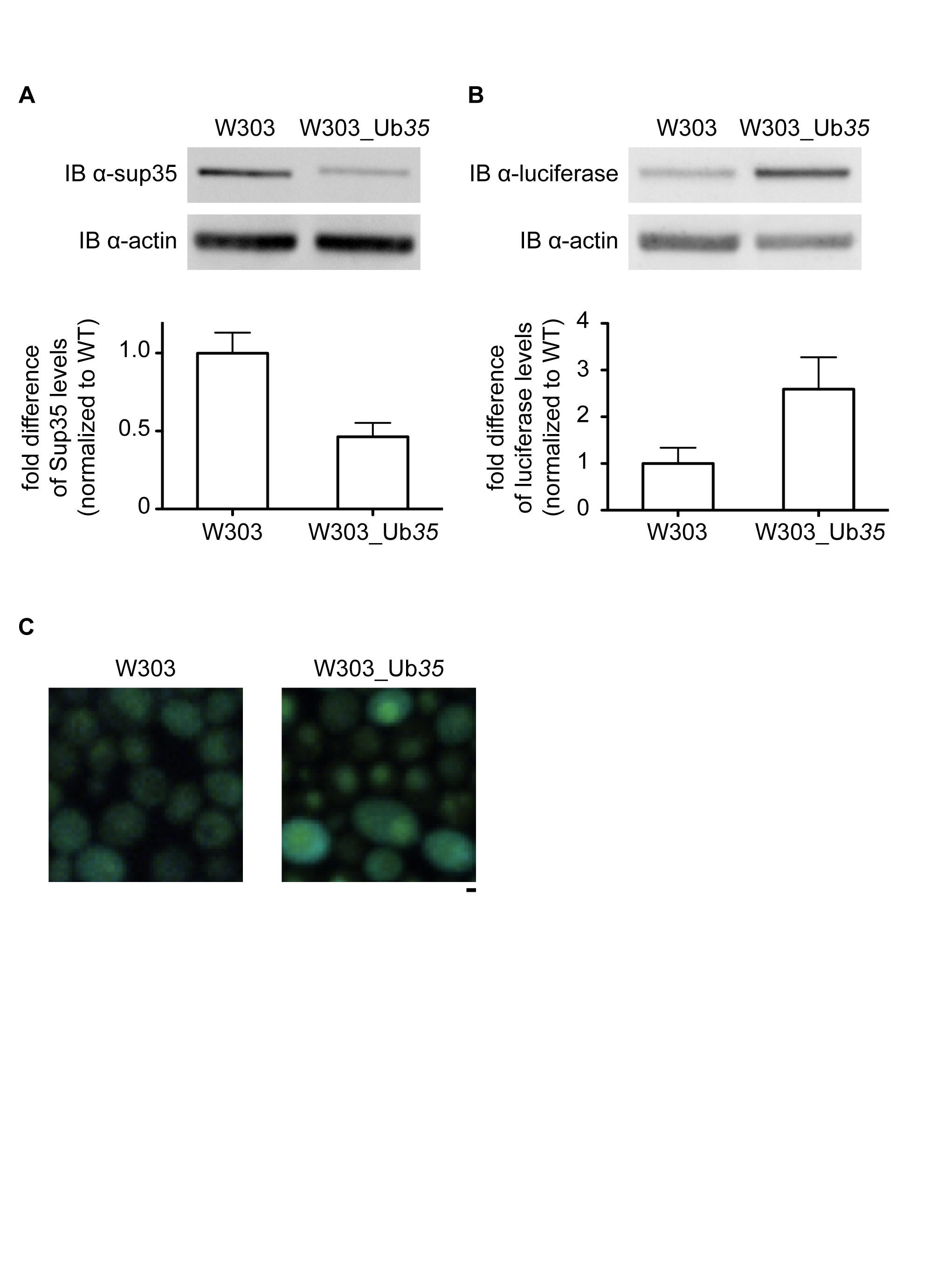

Supplement: Figure S4 — Reduced Sup35 levels in W303_Ub 35 increase the efficiency of amber codon suppression by ANAP incorporation. Immunodetection of Sup35 shows the in vivo concentration of Sup35 in W303_Ub35 is reduced by N-terminal fusion of ubiquitin. Lower panel: Sup35 levels were determined by quantifying western blots using Fujifilm LAS 4000 equipment and Image Reader software. (A) Immunoblotting demonstrates that ANAP incorporation in Luc F161ANAP is improved by destabilization of Sup35 in strain W303_Ub35. Lower panel: Luciferase levels were determined by quantifying western blots using Fujifilm LAS 4000 equipment and Image Reader software. (B) Increased ANAP incorporation efficiency by destabilizing Sup35 in W303_Ub35 analyzed by fluorescence microscopy. Identical microscope settings were used to compare the Luc F161ANAP yield in both strains. Shown are overlays of all 32 channels. Bar, 1 µm. (TIF) [file pone.0099395.s004.tif]
