# Supplementary material for: Monitoring Protein Misfolding by Site-Specific Labeling of Proteins In Vivo
Source: PLoS One. 2014 Jun 10;9(6):e99395. doi: 10.1371/journal.pone.0099395 (PMC4051779; doi:10.1371/journal.pone.0099395)
Supplement: File S1 — This contains Table S1, S2, and S3. Table S1 provides additional information on additional luciferase mutants constructed and analyzed. Table S2 provides the sequences of the DNA oligonucleotides used for luciferase mutant construction and Table S3 provides the sequences of the DNA oligonucleotide used for constructing W303_Ub35 encoding destabilized Sup35. (DOCX) [file pone.0099395.s005.docx]

**Supplementary file S1 contains Supplementary tables S1, S2, and S3.**

| AA position | Residue | Secondary structure | Surface exposed / Buried | Relative activity (WT=100%) |
| --- | --- | --- | --- | --- |
| 15 | Tyr | Random coil | Exposed | 3.4% |
| 127 | Phe | β-sheet | Buried | 0.05% |
| 161 | Phe | Random coil | Exposed | 26% |
| 247 | Phe | α-helix | Buried | 0.02% |
| 273 | Phe | α-helix | Buried | 0.2% |
| 340 | Tyr | β-sheet | Buried | 0.02% |
| 417 | Trp | Random coil | Exposed | 0.2% |
| 465 | Phe | β-sheet | Partially buried | 4.9% |

**Table S1. Analysis of ANAP labeled luciferase mutants.**

| F-QC-luci-15 | 5’-gaaaggcccggtgccaTAGtatccgctagaggatg-3’ |
| --- | --- |
| R-QC-luci-15 | 5’-catcctctagcggataCTAtggcaccgggcctttc-3’ |
| F-QC-luci-127 | 5'-gcagcctaccgtagtgTAGgtttccaaaaaggggt-3' |
| R-QC-luci-127 | 5'-acccctttttggaaacCTAcactacggtaggctgc-3' |
| F-QC-luci-161 | 5'-aacggattaccagggaTAGcagtcgatgtacacgt-3' |
| R-QC-luci-161 | 5'-acgtgtacatcgactgCTAtccctggtaatccgtt-3' |
| F-QC-luci-247 | 5'-tccattccatcacggtTAGggaatgtttactacac-3' |
| R-QC-luci-247 | 5'-gtgtagtaaacattccCTAaccgtgatggaatgga-3' |
| F-QC-luci-273 | 5'-gatttgaagaagagctgTAGttacgatcccttcagg-3' |
| R-QC-luci-273 | 5'-cctgaagggatcgtaaCTAcagctcttcttcaaatc-3' |
| F-QC-luci-340 | 5'-cagggatacgacaaggaTAGgggctcactgagactac-3' |
| R-QC-luci-340 | 5'-gtagtctcagtgagcccCTAtccttgtcgtatccctg-3' |
| F-QC-luci-417 | 5'-gattgacaaggatggaTAGctacattctggagaca-3' |
| R-QC-luci-417 | 5'-tgtctccagaatgtagCTAtccatccttgtcaatc-3' |
| F-QC-luci-465 | 5'-caacaccccaacatcTAGgacgcgggcgtggcag-3' |
| R-QC-luci-465 | 5'-ctgccacgcccgcgtcCTAgatgttggggtgttg-3' |

**Table S2. DNA oligonucleotide sequences used for luciferase mutant construction.**

| F-Ubi | 5’-gcggtttcttcatcgacttgctcggaataacatctatatctgcccactagcaacaATGCAGATTTTCGTCAAG-3’ |
| --- | --- |
| R-Ubi | 5’-ACCACCTCTTAGCCTTAGCACAAGATGTAAGG-3’ |
| F-Ubi-Arg-Sup35FL | 5’-CCTTACATCTTGTGCTAAGGCTAAGAGGTGGTAGATCGGATTCAAACCAAGGC-3’ |
| R-Sup35FL-HA | 5’-ttattaagcgtaatctggaacatcgtatgggtaCTCGGCAATTTTAACAATTTTACC-3’ |
| F-HA-clonNat | 5’-TACCCATACGATGTTCCAGATTACGCTTAATAACGTACGCTGCAGGTCGAC-3’ |
| R-ClonNat-Sup35 | 5’-gaaaatgctttatgatcggtattattgtgtttgcatttacttatgtttgcaagaaatTTAATCGATGAATTCGAGCTCG-3’ |

**Table S3. DNA oligonucleotide sequences used for constructing strain W303_Ub35 encoding destabilized Sup35.**
